# Sinapic Acid Co-Amorphous Systems with Amino Acids for Improved Solubility and Antioxidant Activity

**DOI:** 10.3390/ijms24065533

**Published:** 2023-03-14

**Authors:** Ewa Garbiec, Natalia Rosiak, Ewa Tykarska, Przemysław Zalewski, Judyta Cielecka-Piontek

**Affiliations:** 1 Department of Pharmacognosy, Poznan University of Medical Sciences, 3 Rokietnicka St., 60-806 Poznan, Poland; ewa.garbiec@student.ump.edu.pl (E.G.); nrosiak@ump.edu.pl (N.R.); pzalewski@ump.edu.pl (P.Z.); 2 Department of Chemical Technology of Drugs, Poznan University of Medical Sciences, 6 Grunwaldzka St., 60-780 Poznan, Poland; etykarsk@ump.edu.pl

**Keywords:** sinapic acid, amino acid, lysine, co-amorphous

## Abstract

The objective of this study was to obtain co-amorphous systems of poorly soluble sinapic acid using amino acids as co-formers. In order to assess the probability of the interaction of amino acids, namely, arginine, histidine, lysine, tryptophan, and proline, selected as co-formers in the amorphization of sinapic acid, in silico studies were carried out. Sinapic acid systems with amino acids in a molar ratio of 1:1 and 1:2 were obtained using ball milling, solvent evaporation, and freeze drying techniques. X-ray powder diffraction results confirmed the loss of crystallinity of sinapic acid and lysine, regardless of the amorphization technique used, while remaining co-formers produced mixed results. Fourier-transform infrared spectroscopy analyses revealed that the co-amorphous sinapic acid systems were stabilized through the creation of intermolecular interactions, particularly hydrogen bonds, and the potential formation of salt. Lysine was selected as the most appropriate co-former to obtain co-amorphous systems of sinapic acid, which inhibited the recrystallization of sinapic acid for a period of six weeks in 30 °C and 50 °C. Obtained co-amorphous systems demonstrated an enhancement in dissolution rate over pure sinapic acid. A solubility study revealed a 12.9-fold improvement in sinapic acid solubility after introducing it into the co-amorphous systems. Moreover, a 2.2-fold and 1.3-fold improvement in antioxidant activity of sinapic acid was observed with respect to the ability to neutralize the 2,2-diphenyl-1-picrylhydrazyl radical and to reduce copper ions, respectively.

## 1. Introduction

Phenolic acids are a group of secondary plant metabolites belonging to polyphenol compounds. They contain phenolic ring and a carboxylic acid function and are derivatives of hydroxycinnamic and hydroxybenzoic acids [1]. Phenolic acids have many health-promoting properties and are essential components of the human diet [2]. Particularly valuable for its beneficial health properties is sinapic acid (3,5-dimethoxy-4-hydroxycinnamic acid, SA). It is present in high concentrations especially in oilseeds and vegetables belonging to the *Brassicaceae* family [3]. It possesses a wide range of properties resulting from anti-inflammatory and antioxidant activity. SA can exert antioxidant effects, primarily as a free radical scavenger. It is, therefore, a promising compound in the prevention and treatment of many disorders, whose development is associated with oxidative stress. It was proven, that SA can causes neuroprotective [4], hepatoprotective [5], nephroprotective [6], and gastroprotective [7] effects.

Phenolic acids are often poorly soluble [8,9,10,11], while solubility can be a parameter that influences oral bioavailability [12]. Literature data on the solubility of SA are lacking. Demurtas et al. [13] determined that SA is the least soluble among other hydroxycinnamic acids (caffeic, ferulic, o-coumaric, m-coumaric, and p-coumaric acid). Thus, it is possible that SA’s potential therapeutic effects may be limited by its low bioavailability, resulting from its poor solubility.

Therefore, work on improving the solubility of SA is warranted. Sinha et al. [14] obtained co-crystals of SA with nicotinamide, while Ahad et al. [15] prepared an inclusion complex of SA with hydroxypropyl-β-cyclodextrin (HPβCD) by means of a solvent evaporation technique. With the addition of HPβCD, the solubility of SA improved. In the presence of HPβCD, the aqueous solubility of another hydroxycinnamic acid, *trans*-ferulic acid, was also significantly increased [9]. Various other techniques and methods have been used to improve the water solubility of phenolic acids, for example, the preparation of electrospun nanofibers [16,17], encapsulation using spray drying [18], and amorphous solid dispersions [19].

Amongst the abovementioned methods, a recognized technique for increasing solubility is also amorphization [20]. The term “amorphization” refers to the loss of a long-range crystalline structure. Amorphous forms are characterized by enhanced solubility compared to their crystalline counterparts. However, they are thermodynamically unstable and show a tendency to recrystallize [21]. Therefore, to increase physical stability, excipients called co-formers are used. Amino acids, as low molecular weight, and generally recognized as safe (GRAS) compounds, are of particular interest in this respect [22,23]. Amino acids contain α-carboxylate and α-amino groups, allowing them to interact as hydrogen acceptors or donors and form molecular interactions with some active pharmaceutical ingredient (API) [24].

The aim of this study was to define the interactions between SA with selected amino acids and to evaluate the effect of the obtained co-amorphous systems on its solubility and antioxidant potential.

## 2. Results

This research’s objective was to obtain co-amorphous systems of SA with amino acids and to select the optimal amino acid based on the results of X-ray powder diffraction (XRPD) as a reference method and with the support of molecular modeling. The SA systems with the selected amino acid were characterized by Fourier-transform infrared spectroscopy (FT-IR), scanning electron microscopy (SEM), and thermogravimetric (TG) analysis. In addition, their physical stability, solubility, and dissolution rate were evaluated, as well as their biological properties with respect to antioxidant activity.

Co-amorphous formulations of SA with amino acids were obtained by using ball milling, solvent evaporation, and freeze drying methods. The binding energy between SA and the chosen amino acids was calculated using molecular modeling in order to find the best amino acid co-formers. It has also been demonstrated that hydrogen bonds can form between the oxygen and hydrogen atoms of SA and those of selected amino acids. Summarized values of the binding energy of SA systems with lysine (LYS), tryptophan (TRP), arginine (ARG), histidine (HIS), and proline (PRO) are presented in Table 1. An optimized geometry of all structures, 3D and 2D interactions of SA with amino acids, are shown graphically in the Supplementary Materials (Figure S1).

### 2.1. Identification of Sinapic Acid—Amino Acids Systems

#### 2.1.1. X-ray Powder Diffraction

**Figure 1:**
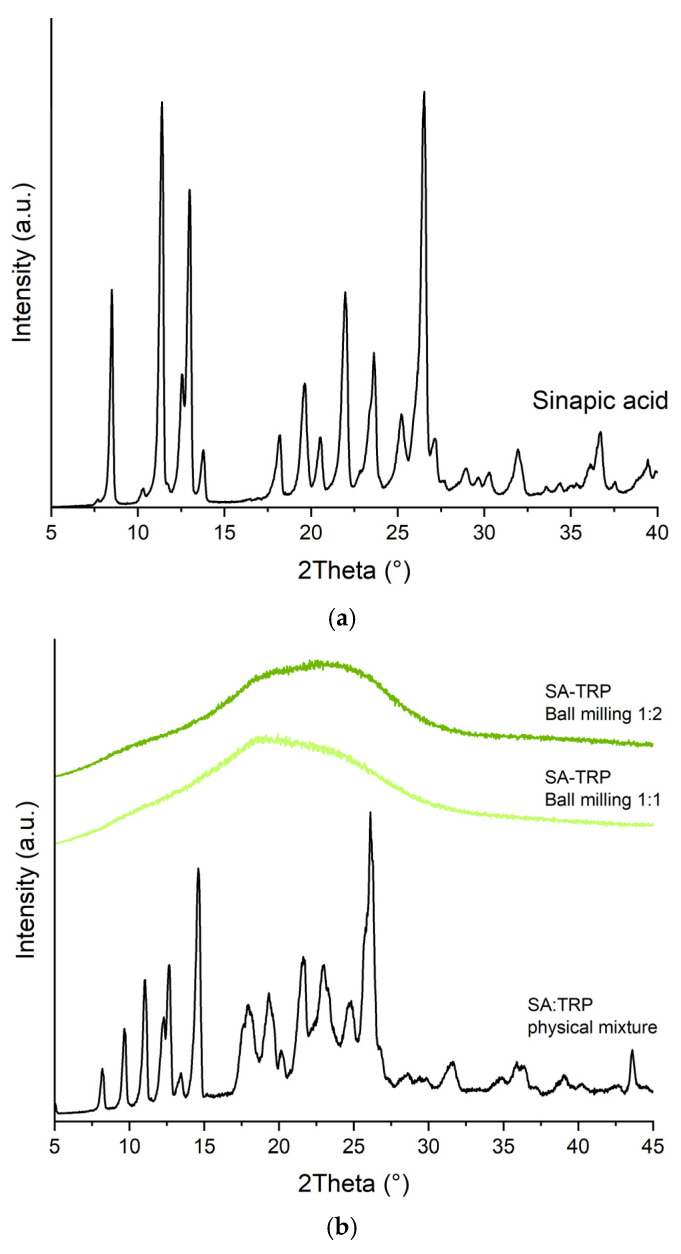

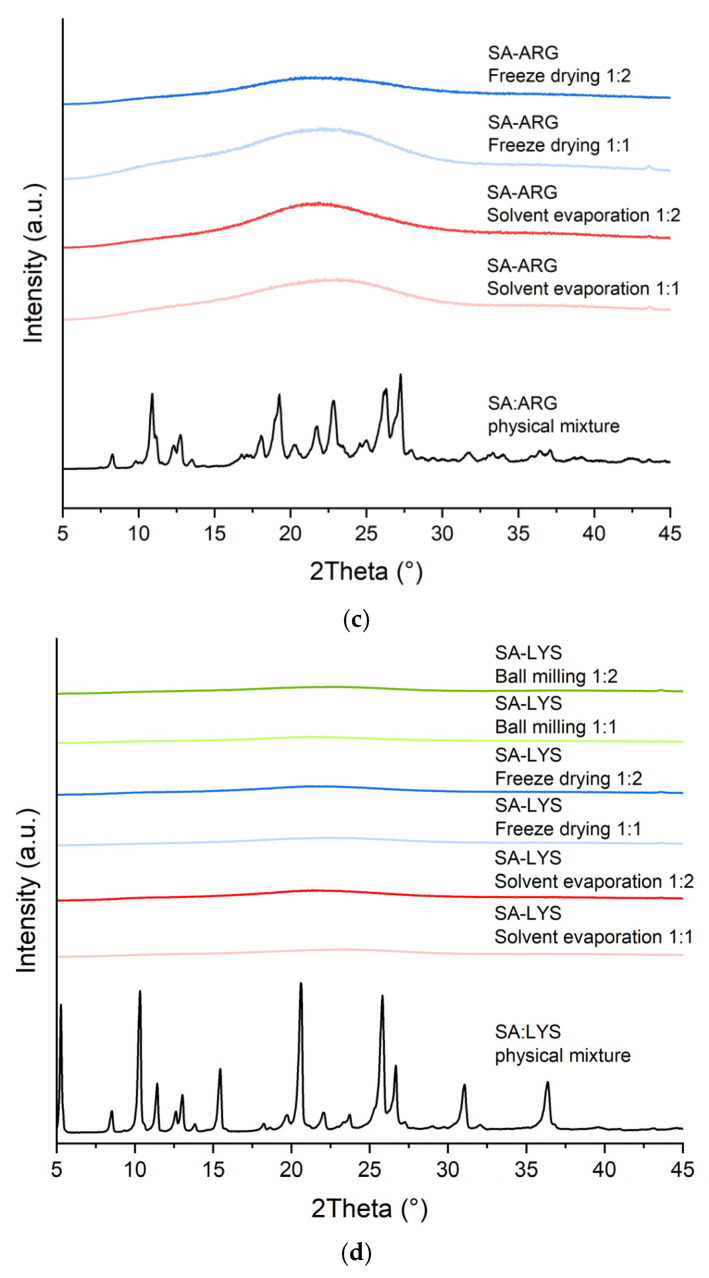
The XRPD diffraction patterns of SA (**a**), co-amorphous systems of SA with TRP and the physical mixture (**b**), co-amorphous systems of SA with ARG and the physical mixture (**c**), co-amorphous systems of SA with LYS and the physical mixture (**d**).

SA has a crystalline structure and long-range ordering, observed as sharp diffraction peaks at 2θ of 8.5°, 11.4°, 12.9°, 13.7°, 18.2°, 19.6°, 20.5°, 21.9°, 25.2°, 26.5°, 31.9°, and 36.7° (Figure 1a). The crystallinity of SA is also visible in its physical mixtures with amino acids. SA has become fully amorphous in the systems with LYS, which is indicated in all XRPD diffractograms by the “halo effect”. This term refers to a broad peak that appears in the diffraction pattern of amorphous materials, that do not have a long-range ordered structure, in contrast to crystalline materials that produce sharp, well-defined peaks in their diffraction patterns. LYS, therefore, enabled the amorphization of SA by all the methods used in both molar ratios (Figure 1d). TRP made it possible to obtain amorphous SA systems in both molar ratios (1:1 and 1:2) only by ball milling technique, while ARG was important in obtaining them as a result of the evaporation process and freeze drying in both molar ratios (Figure 1b,c). HIS and PRO exhibited poor co-formability upon used preparation techniques as their characteristic peaks remain detectable in XRPD diffraction patterns. Moreover, it is worth noting that none of the methods applied made SA convert to the amorphous form without the addition of the co-former. Future characterization and studies were, therefore, conducted for systems of SA with LYS.

#### 2.1.2. Scanning Electron Microscopy

**Figure 2:**
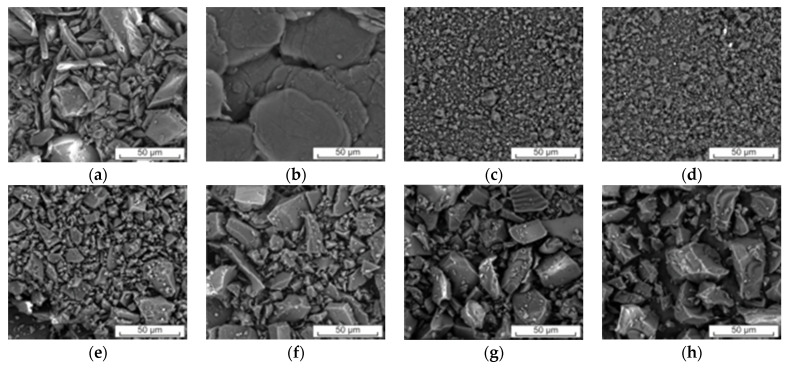
Scanning electron microscopy images of SA (**a**), LYS (**b**), co-amorphous system of SA with LYS in molar ratio 1:1 obtained by ball milling (**c**), co-amorphous system of SA with LYS in molar ratio 1:2 obtained by ball milling (**d**), co-amorphous system of SA with LYS in molar ratio 1:1 obtained by solvent evaporation (**e**), co-amorphous system of SA with LYS in molar ratio 1:2 obtained by solvent evaporation (**f**), co-amorphous system of SA with LYS in molar ratio 1:1 obtained by freeze drying (**g**), co-amorphous system of SA with LYS in molar ratio 1:2 obtained by freeze drying (**h**).

In all cases, a reduction in size and shape occurred. SEM observations showed that initial SA (Figure 2a) and LYS (Figure 2b) particles are different in shape. SA particles are angular, and LYS are flaky. Moreover, SA powder is a mixture of coarse particles with sizes of <10 µm and >50 µm, in contrast to LYS where only coarse particles with a size of ≈75 µm are visible. What is worth noticing, nodular particles obtained after ball milling are fine-grained with a size of <1 µm and coarse-grained with a size of >10 µm (Figure 2c,d). The systems obtained by solvent evaporation (Figure 2e,f) are characterized by a bimodal powder size with angular particles of sizes 1–5 µm and 20–40 µm. Systems obtained by freeze drying (Figure 2g,h) are also characterized by a bimodal powder size but with particle sizes of ≈10 µm and ≈50 µm. Systems obtained by all methods exhibited morphological changes compared to initial SA and LYS powders.

#### 2.1.3. Fourier-Transform Infrared Spectroscopy

FT-IR analyses were performed to identify the main bands of compounds and investigate potential interactions between SA and LYS. The FT-IR spectrum of the physical mixtures showed no changes, which are, however, observed in the spectra of co-amorphous systems. The FT-IR spectra of the co-amorphous systems of SA-LYS, physical mixtures, and the individual components are found in the Supplementary Materials (Figures S5 and S6), as well as the optimized geometry of the SA and LYS (Figures S3a and S4a, respectively). By using quantum chemical calculations, it is possible to identify which specific functional groups of a molecule correspond to the observed bands in experimental spectra. The experimental findings were then compared to the calculations (Figures S3b,c and S4b,c). The most important bands (theoretical and experimental) of the SA and LYS are collected in Table S1 (see Supplementary Materials).

The most intense bands of the crystalline SA were observed in the FT-IR spectrum at about 800–1700 cm^−1^ and 2400–3400 cm^−1^. For example, the range between 2400 and 3400 cm^−1^ is dominated by bands related to the stretching vibration of the O–H and C–H bonds. Next, the range of about 1700–1350 cm^−1^ predominates the bands corresponding to the C=O, C–O–H, and C–C–C.

The most characteristic bands of the crystalline LYS are in the range of 2600–3400 cm^−1^ (NH_2,_ CH_2_ and C–H stretching vibration), 1600–1150 cm^−1^ (predominate the bands corresponding to the C=O, C–O–H, and NH_2_), and 1000–500 cm^−1^ (predominate the bands corresponding to the C–O–H, C–C–C, C–H, and O–H).

The FT-IR spectra of the 1:1, as well as 1:2 co-amorphous systems obtained by the ball milling, solvent evaporation, and freeze drying method are identical. The main changes that indicate the amorphous state of SA in the systems in molar ratio 1:1, are (1) disappearance of characteristic peaks of SA (814 cm^−1^, 993 cm^−1^, and 1657 cm^−1^); (2) shifting and/or changing the shape of the peaks (SA) in the range 800–1700 cm^−1^; (3) shifting and decrease in the intensity of the peaks in the range 2800–2950 cm^−1^ (C–H stretching in SA and LYS); (4) disappearance of peaks corresponding to the O–H stretching in SA and N–H stretching in LYS (3377 cm^−1^ and 3306 cm^−1^). In the systems in molar ratio 1:2, the main changes that indicate the amorphous state of SA are (1) the disappearance of characteristic peaks of SA (814 cm^−1^, 872 cm^−1^, 907 cm^−1^, 993 cm^−1^, 1591 cm^−1^, 1620 cm^−1^, and 1657 cm^−1^); (2) shifting and/or changing the shape of the peaks (SA) in the range 800–1520 cm^−1^; (3) shifting and decrease in the intensity of the peaks in the range 2800–2950 cm^−1^ (C–H stretching in SA and LYS); (4) disappearance of peaks corresponding to the O–H stretching in SA and N–H stretching in LYS (3306 cm^−1^ and 3377 cm^−1^).

#### 2.1.4. Thermogravimetric and Differential Scanning Calorimetry

TG analysis confirmed that SA is thermally stable up to about 170 °C, while LYS is stable up to about 215 °C. The differential scanning calorimetry (DSC) thermogram of SA revealed a sharp crystalline peak at a melting temperature of 193 °C (see Figure S8, dashed black line, Supplementary Materials). The obtained co-amorphous systems exhibited a decrease in thermal stability (see Figure S8, Supplementary Materials).

TG analysis provided evidence for changes in the thermal stability of SA and LYS in the obtained systems. The first thermal effect observed in all obtained systems suggests water loss. Subsequent effects are related to the thermal decompositions of the samples. Decomposition at about 193 °C is around 24% (*w*/*w*).

### 2.2. Physical Stability

Co-amorphous systems were stored at 30 °C and 50 °C under uncontrolled humidity. To characterize the solid-state forms of the samples after 2, 4, and 6 weeks of storage, XRPD was used (Figure S2, Supplementary Materials).

Physical stability studies showed that LYS was effective in stabilizing co-amorphous systems of SA obtained by all methods used, at both molar ratios, as results obtained by XRPD showed no recrystallization peaks during six weeks of storage. There were also no differences in the physical appearance of the samples. All systems were stable up to the end of the study at both temperatures.

### 2.3. Solubility Study

The solubility study results in distilled water and HCl 0.1 N of pure SA and the co-amorphous systems of SA with LYS obtained by all methods used, in molar ratio 1:1, are presented in Table 2. Pure SA solubility was found to be 0.226 ± 0.001 mg·mL^−1^ and 0.209 ± 0.005 mg·mL^−1^ in water and in HCl 0.1 N, respectively. Co-amorphous systems with LYS as a co-former showed significant improvement in solubility compared with pure SA. The ball milled system showed the greatest increase in solubility in both media, which was 12.9-fold in water and 1.5-fold in HCl 0.1 N. There were no statistically significant differences between systems obtained using different methods in terms of improving solubility in HCL, while in water the evaporation-obtained system enabled an almost 12-fold solubility rise, and the system obtained by freeze drying achieved an about 11-fold increase in solubility.

### 2.4. Dissolution Rate Studies

**Figure 3:**
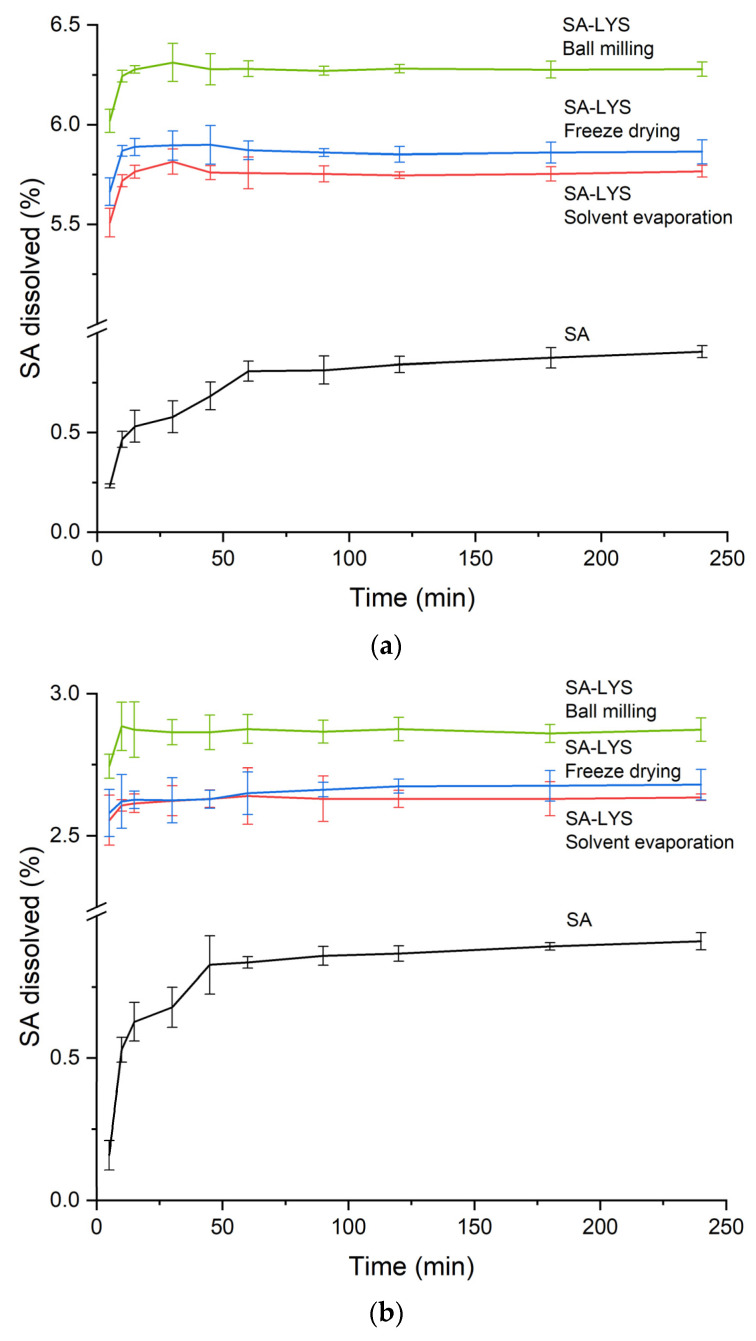
Dissolution profiles of SA and SA co-amorphous systems with LYS in molar ratio 1:1 in water (**a**) and HCl 0.1 N (**b**).

Dissolution profiles of pure SA and obtained systems are depicted in Figure 3a,b. At the end of the study, pure SA reached the maximum dissolution of about 0.9% both in the water and in HCl 0.1 N. A comparable trend of SA release from the obtained systems was observed in both media; however, the dissolution rate was greater in water. The highest increase was observed in the system obtained by the ball milling method and was found to be enhanced approximately 7-fold in water and 3-fold in HCl 0.1 N in comparison to crystalline SA. In sum, a significantly higher than pure SA dissolution rate was observed for all systems, in both media, and the supersaturation achieved was maintained throughout the study.

### 2.5. Antioxidant Activity

**Figure 4:**
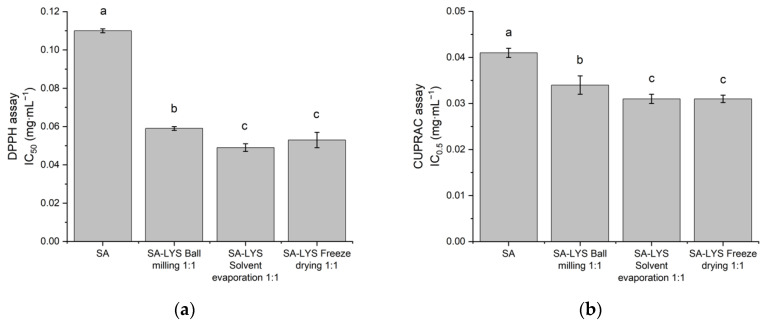
Antioxidant activity of SA and SA co-amorphous systems with LYS in molar ratio 1:1 evaluated using the DPPH (**a**) and CUPRAC (**b**) models. Mean values with the same letter are not significantly different at *p* < 0.05 using Duncan’s multiple range test. “a” stands for the highest values, “b” and “c” stand for the statistically significant decreasing values.

The antioxidant activity of SA introduced into the co-amorphous systems was assessed using DPPH and CUPRAC methods and expressed as IC_50_ and IC_0.5_, respectively. In both assays, elevated antioxidant activity, with a similar pattern, was observed for co-amorphous SA systems—the best results were achieved for the systems obtained by solvent evaporation and the freeze drying method. There were no statistically significant differences between those methods. A 2.2-fold improvement in SA antioxidant activity was shown for the systems obtained by the solvent evaporation method for the DPPH assay (reaching an IC_50_ of 0.049 ± 0.002 mg·mL^−1^) and a 1.3-fold improvement for the CUPRAC assay (with IC_0.5_ equal 0.031 ± 0.001 mg·mL^−1^) (see Figure 4).

## 3. Discussion

SA is a promising compound with a wide range of various bioactive properties resulting from its antioxidant activity. Those properties are, however, limited because of its poor solubility. A promising approach to overcome this limitation is to transform a substance from a crystalline to an amorphous state. Our aim was to obtain co-amorphous systems of SA with selected amino acids, choose the optimal co-former and assess the impact on the solubility and antioxidant properties.

Co-amorphous systems of SA with amino acids were prepared by means of ball milling, solvent evaporation, and freeze drying. All of these methods are recognized means for obtaining amorphous formulations [22,24,25,26]. They are considered gentle preparation methods, also suitable for heat labile compounds such as amino acids. These methods rely on different mechanisms of action since grinding involves a disordering of the crystal structure through mechanical disruption and is a solvent-free preparation technique, in contrast to the freeze drying and solvent evaporation methods. However, it was not noted that there was a dependence on the type of method used on a possible successful co-amorphization of SA. The type of amino acid used had a greater influence on achieving amorphization than the method applied (see Table 3).

The most successful in achieving co-amorphization was LYS, as a broad halo pattern in all XRPD diffractograms confirmed the amorphous state of SA. Furthermore, by performing FT-IR analysis, the interactions between SA and LYS were defined. As opposed to the crystalline counterpart, amorphous SA in the obtained systems showed wider and less strong peaks as well as the disappearance of many characteristic peaks. The observed changes suggest the formation of amorphous salts and the presence of intermolecular hydrogen bonds. The stretching vibration of the –COOH group of SA observed at 3377 cm^−1^ disappeared in SA-LYS systems, indicating that deprotonation has occurred in the –OH group of SA. In the range of 3200–3400 cm^−1^, peaks corresponding to the NH_2_ asymmetric stretching in the side chain of LYS (3356 cm^−1^) and NH_2_ stretching in the amine group of LYS (3291 cm^−1^) in the SA-LYS systems disappeared completely. A peak at about 1620 cm^−1^ (C=O stretching vibration in SA) in the systems was recorded at about 1636 cm^−1^, while other peaks corresponding to the C=O group of SA (1657 cm^−1^) completely disappeared. Changes in the C=O groups could be attributed to the restriction with the –NH group of LYS. Analogous changes in the FT-IR spectra of the salt were observed by Yu et al. for caffeic acid with venlafaxine [27]. Losing a proton indicates ionization, which can lead to the creation of salt [28,29,30]. The literature shows that amino acids have the ability to produce salts [31,32]. Salt formation is largely influenced by the pK_a_ difference, and if the difference is greater than 2, salt creation is more likely to occur [30,33]. The pK_a_ difference between SA and LYS is 6.3, which implies that complete salt formation is possible. Therefore, the FT-IR analysis findings are in line with the literature.

The formation of hydrogen bonds between SA and LYS was confirmed by the main changes observed in the co-amorphous systems concerning the peaks corresponding to C–O–H in the carboxyl group (725, 1335, 1516, 1620 and 1657 cm^−1^), O–H (907, 1115, 1157, 1215, 1267, 1294, 1387, 3306 and 3377 cm^−1^), C–O (1115 and 1516 cm^−1^), C–O–C (972 cm^−1^) of SA, and the NH_2_ group (3356 and 3291 cm^−1^) of LYS.

The experimental study’s results are also consistent with the theoretical predictions, which indicated the possibility of an interaction between SA and LYS. With the lowest binding energy (–1.71 kcal·Mol^−1^) among the selected amino acids, LYS was chosen as the most energetically favored co-former. The good co-formability properties of LYS have also been reported in the literature. There are several studies in which co-amorphous systems with LYS were obtained. After ball milling with LYS, mebendazole, simvastatin, and furosemide were fully converted to co-amorphous systems [34]. Indomethacin became co-amorphous with LYS upon ball milling [34,35] and spray drying [36,37].

Concerning ARG, it showed overall good co-formability and led to the amorphization of SA by means of solvent-based methods: freeze drying and solvent evaporation. There are several studies that successfully used ARG as a co-former in co-amorphous drug systems. Hatwar et al. [38] obtained a co-amorphous system with quercetin by the ball milling method. Quercetin incorporated in pellets produced on their basis showed enhanced solubility and bioavailability. In our study, ARG did not produce a co-amorphous system with SA in the ball milling process. This may result from using a shorter, 1 h milling time (quercetin was ground with ARG for 2 h). Lenz et al. [39] have developed a tablet formulation of indomethacin with ARG obtained by spray drying. Tablets indicated physical stability during storage, and the dissolution behavior of API was improved. Khanfar et al. [24] prepared co-amorphous systems of telmisartan with ARG by means of freeze drying. They pointed out the relationship between the molar ratio and the characteristics of the obtained systems. The 1:0.5 telmisartan-ARG ratio system showed the highest physical stability, while the 1:2 system had the highest solubility.

Within the group of non-polar amino acids, only TRP produced co-amorphous systems, and only in the ball milling process. However, these systems were characterized by limited physical stability, as recrystallization peaks were present in the second week of storage. Moreover, no improvement in solubility nor in antioxidant activity was observed. Attempts to acquire co-amorphous systems with TRP through the use of solvent-based techniques resulted in separation and precipitation of TRP from the solution. Regarding PRO, it did not allow the amorphization of SA in any of the methods used. PRO alone was also found to be not sufficient to achieve amorphization of naproxen in a study carried out by Jensen et al. [31]. Only the addition of a second amino acid (TRP or ARG) enabled the obtainment of co-amorphous formulations of naproxen. It is suggested that PRO is difficult to convert to the amorphous form due to the high rigidity of its molecule. However, according to the screening method of choosing amino acids for co-amorphous formulations, developed by Kasten et al. [34], PRO is considered as a generally suitable co-former for acidic compounds, such as SA. It is possible to suggest that changes in the milling process parameters, such as milling frequency or increasing the grinding time, would result in amorphization. Kasten et al.’s study also indicates that good co-formers for acidic drugs are basic amino acids, because of differences in pK_a_ and possible amorphous salt formation. SA pK_a_ is 4.5 [40], ARG pK_a_ = 12.5, LYS pK_a_ = 10.8, and HIS pK_a_ = 6.0. The required difference in pKa values (>2 or 3) for the formation of amorphous salts between co-formers and SA is, therefore, preserved for ARG and LYS. For HIS, it is equal to ΔpK_a_ = 1.5, and HIS indeed failed to produce co-amorphous systems with SA in our study. Considering the aforementioned outcomes, further studies were conducted for the SA-LYS co-amorphous systems.

Thermal properties of SA, LYS, and co-amorphous systems of SA with LYS were determined using a TG and DSC analysis. The melting point temperature of SA was found to be 193 ℃, which is in agreement with the previously reported value [41]. According to thermogravimetric analysis results, the obtained co-amorphous systems exhibited around 24% (*w*/*w*) mass loss at 193 °C; therefore, it was not possible to conduct a DSC analysis up to a temperature that would allow confirming the disappearance of the melting point of SA.

As a known drawback of amorphous systems is their tendency to recrystallize, the co-amorphous formulations of SA with LYS were subjected to stability studies. Amorphous materials tend to absorb water into the inside of the system, so they can strongly absorb moisture from the air. Water lowers the glass transition temperature (T_g_), has a plasticizing effect, and can lead to recrystallization [42]. It was noticed that upon storage at humidity levels equal to 65% and 75%, co-amorphous formulations got melted. Therefore, only the influence of temperature on the stability of the co-amorphous formulations of SA with LYS was assessed. LYS was found in our study to be a promising co-former for SA with regard to physical stability. All of the obtained systems were considered stable, as no recrystallization occurred at any of the used temperatures (30 °C and 50 °C) during six weeks of storage. No differences were noted with regards to the techniques used, nor the molar ratio. LYS is, therefore, considered as a promising co-former to improve the amorphous stability of SA. The physical stability of the formulations using LYS as a co-former was also reported by Kasten et al. [35], who compared crystalline and co-amorphous forms of salts of indomethacin-LYS. For the entire period of the study (36 weeks, at 25 °C and 40 °C, 2% RH), they did not observe the appearance of recrystallization peaks in the XRPD pattern.

In order to assess how the solubility of SA was affected after its introduction into the co-amorphous systems, the solubility study was conducted. The assay was performed for systems in a molar ratio of 1:1, since our study showed that the optimal relationship between SA and LYS is an equimolar ratio, as SA’s chemical stability in the solution is preserved in this proportion. The water solubility of SA was determined to be 0.226 ± 0.001 mg·mL^−1^, which classifies SA as very slightly soluble [43]. Determining the solubility of SA in water by means of the HPLC method is an additional value of our work, as the literature is lacking such data. Co-amorphous systems of SA with LYS obtained in the ball milling process have led to a 12.9-fold increase in solubility. The solubility of SA in HCl 0.1 N was lower and reached 0.209 ± 0.005 mg·mL^−1^, while the system obtained by the ball milling method allowed its 1.5-fold improvement. Considering this, SA’s solubility appears to be pH-dependent, which is presumably attributed to its weak acid character. The improvement in solubility, in addition to the amorphous nature of the substance, can, therefore, also be enhanced by the presence of LYS, which is highly soluble and, when dissolved, can change the pH of the layer surrounding SA [42]. At a higher pH, however, SA is known to undergo oxidative conversion which may result in a decomposition, leading to the formation of 6-hydroxy-5,7-dimethoxy-2-naphthoic acid and 2,6-dimethoxy-p-benzoquinone [44]. Alkaline pH is, therefore, not a suitable environment in order to evaluate SA’s solubility.

The obtained systems remarkably enhanced SA solubility compared to results from the literature. Ahad et al. [15] reported an about 2.59-fold improvement in the solubility of SA in water in the presence of 10 mM HPβCD. In another study, SA solubility was determined to be 0.0028 M and it increased to 0.2 M for its corresponding cholinium salt [13].

To assess the dissolution behavior of the obtained co-amorphous systems in a water and acidic environment, a dissolution rate study was performed. All the systems obtained presented a dissolution advantage compared to crystalline SA, the highest of which represented an almost 7-fold enhancement in water for the system prepared by means of ball milling. This particular system exhibited also the most significant enhancement in the solubility assay. Considering this, and the observation that improvement in solubility and apparent solubility was less pronounced in HCl 0.1 N for all the systems produced, it can be concluded that the findings of the solubility and dissolution studies are consistent. Such a relationship has also been previously reported in the literature [24,45].

It is well known that amorphous forms are able to generate supersaturated solutions [30,39]. In order to benefit from increased apparent solubility, it is necessary to maintain the highest possible level of supersaturation [46]. Co-amorphous SA systems obtained by all methods, after a quick plateau achievement, allowed it to be maintained until the end of the study. This may have been influenced by the interaction between SA and LYS, which have translated into a reduced risk of recrystallization. The presence of such interactions in the obtained co-amorphous systems was indicated by the results of FT-IR analysis. The abovementioned finding is supported by literature data according to which the occurrence of interactions, particularly the potential for salt formation, may be linked to the improvement in dissolution rate and preservation of the maximum supersaturation [47,48]. The obtained results also support literature findings on the usefulness of LYS in creating and sustaining drug supersaturation in co-amorphous systems. The co-amorphous salt of indomethacin with LYS produced by Kasten et al. translated into a 35.2-fold increase compared to the dissolution rate of the crystalline drug counterpart, and allowed supersaturation to be maintained for 6 h [35].

The health-promoting properties of SA are mainly due to its ability to scavenge free radicals. To evaluate the antioxidant potential of a sample, a single assay is not sufficient. The antioxidant activity of SA was tested in DPPH and CUPRAC assays, as both methods are suitable for assaying the antioxidant activity of phenolic acids [49,50]. CUPRAC is an electron transfer-based (ET) assay, while the DPPH assay is considered to be the frontier between an ET- and hydrogen atom transfer (HAT)- assay [51]. The DPPH radical can either accept an electron or receive a hydrogen atom. According to the literature [13], in the presence of the DPPH radical, the possible mechanism of free radicals scavenging by SA may occur via HAT or a sequential proton loss electron transfer (SPLET) mechanism. In our study, crystalline SA showed the ability to scavenge free radicals in the DPPH assay, with IC_50_ equal 0.110 ± 0.001 mg·mL^−1^ and was confirmed to cause a reduction of copper ions; its IC_0.5_ was determined to be 0.041 ± 0.001 mg·mL^−1^. SA, in the obtained co-amorphous systems with LYS, was demonstrated to have an enhanced antioxidant activity. The highest antioxidant activity was observed for the systems obtained by the solvent evaporation and freeze drying method in both assays (in which the DPPH IC_50_ is 0.049 ± 0.002 mg·mL^−1^ and 0.053 ± 0.004 mg·mL^−1^ and the CUPRAC IC_0.5_ is 0.031 ± 0.001 mg·mL^−1^ and 0.032 ± 0.0008 mg·mL^−1^, for solvent evaporation and lyophilization, respectively). The improvement in the antioxidant properties of SA in the systems obtained is most likely related to its solubility improvement. These results are supported by the studies of Ahad et al. [15] who observed a stronger DPPH radical scavenging activity for the obtained inclusion complex of SA with HPβCD compared to pure SA, and concluded that antioxidant activity can be enhanced as a result of solubility improvement. However, it is not excluded that, in addition to the effect of solubility improvement, the presence of LYS in the co-amorphous systems also had an impact on the observed antioxidant activity improvement. Due to their structure, amino acids can exhibit antioxidant properties, whose underlying mechanisms vary due to structural differences in their side chains [52]. In addition, those properties can be changed upon bonding to metal ions [53,54,55]. According to the literature [56], LYS exhibits ferrous ion-chelating activity and DPPH radical- and hydroxyl radical-scavenging activity, among others. However, with respect to our systems, this effect seems to be of negligible importance—in the CUPRAC assay, the concentration of LYS needed to reach an IC_0.5_ value was nearly 23 times greater than the concentration of LYS present in the system being studied; whereas when it comes to the DPPH assay, LYS failed to achieve the IC_50_ value, which is in line with a report from the literature [56] (maximum radical inhibitory activity in our study was equal to 38.8% for the LYS concentration more than twice of the concentration in the studied system). Taking into account these considerations and the reports from the literature indicating that an increase in the solubility of the compound may translate into antioxidant activity improvement [45,57], we correlated an increase in SA properties in the DPPH and CUPRAC assays mainly with the solubility improvement.

## 4. Materials and Methods

### 4.1. Materials

SA (purity ≥ 98%), ARG (purity of 99%), LYS (purity ≥ 98%), neocuproine (purity ≥ 98%), 2,2-diphenyl-1-picrylhydrazyl, were delivered from Sigma-Aldrich (St. Louis, MO, USA). HIS (purity > 99%) and TRP (purity > 98.5%) were supplied by TCI Chemicals (Portland, OR, USA). PRO (purity ≥ 98.5%) was provided by Carl Roth Gmbh & Co. KG (Karlsruhe, Germany). The ammonium acetate pure and analytical weighed amount hydrochloric acid 0.1 N was supplied by Chempur (Piekary Śląskie, Poland). Methanol (HPLC grade), ethanol, and copper (II) chloride dihydrate pure were delivered from POCH (Gliwice, Poland) and acetic acid from Avantor Performance Materials Poland S.A. (Gliwice, Poland).

### 4.2. Preparation of Co-Amorphous Systems

#### 4.2.1. Ball Milling

A total of 600 mg of a physical mixture consisting of SA and amino acids in a molar ratio of 1:1 or 1:2 was ground in a ball mill, Mixer Mill MM400 (Retsch GmbH, Haan, Germany). Samples were milled at a frequency of 30 Hz, in a 25 mL jar containing two stainless steel balls with a diameter of 10 mm, for 12 min, then cooled for 7 min. This was repeated for 5 cycles.

#### 4.2.2. Solvent Evaporation

After the dissolution of 600 mg of a physical mixture consisting of SA and amino acids in a molar ratio of 1:1 or 1:2 in the mixture of methanol:water 30:70 (*v*/*v*), the solvent was removed under reduced pressure using a rotary evaporator (Rotavapor R-210, Buchi Corp., New Castle, DE, USA) with a water bath maintained at 40 °C. Solvents were chosen based on the solubility of SA and amino acids.

#### 4.2.3. Freeze Drying

A total of 600 mg of a physical mixture consisting of SA and amino acids in a molar ratio of 1:1 or 1:2 were dissolved in the mixture of methanol:water 30:70 (*v*/*v*). Pre-frozen samples were freeze dried under the conditions of −55 °C, 6 hPa for 24 h in a lyophilizer (Heto PowerDry PL3000) Freeze Dryer (Thermo Scientific, Waltham, MA, USA). Solvents were chosen based on the solubility of SA and amino acids.

### 4.3. Molecular Modeling

#### 4.3.1. Structure for Molecular Docking

The SA and amino acids’ (ARG, HIS, LYS, PRO, and TRP) structures were retrieved from the PubChem database in SDF format (website: https://pubchem.ncbi.nlm.nih.gov/, accessed on 10 May 2021).

#### 4.3.2. OPEN Babel GUI

Using Open Babel (the Open Babel Package, version 2.4.0, http://openbabel.org, accessed on 11 May 2021), SA and amino acids’ SDF files were converted into MOL2 and PDB formats.

#### 4.3.3. Molecular Docking Using AutoDock 4.2.6 Software

AutoDock 4.2.6 was downloaded from ‘The Scripps Research Institute’ official website (website: http://autodock.scripps.edu/, accessed on 15 February 2021).

#### 4.3.4. Initializing and Preparation of PDBQT Files

Before docking, the starting directory was set to the desired folder. The SA molecule (in MOL2 format) was loaded using the AutoDock Tool’s ligand menu. The torsion tree was defined by choosing the root; the number of rotatable bonds was identified, and the file was saved in PDBQT format. The amino acid (ARG, HIS, LYS, PRO or TRP, in PDB format) was imported into the workstation; polar hydrogen atoms were added; the Kollman and Gasteiger charges were computed for the amino acid. Next, the file was saved in PDBQT format that was then used as the target. The SA and amino acid were imported in PDBQT format into the workspace for a further simulation process.

#### 4.3.5. Grid Parameters

Docking was carried out using AutoDock Vina, by using the PDBQT files of the SA, amino acid, and the configuration file containing the dimensions of the grid box. Grid spacing and the center grid box values were set to default. The number of grid points along the x, y, and z dimensions were set as 40 × 40 × 40. The total grid points per map was 64,000. The output was saved in the grid parameter file (GPF) file format.

#### 4.3.6. Running AutoGrid and AutoDock

Following the creation of GPF files, the AutoGrid was run with the GPF files as an input, and it transformed them into grid log files (GLG). After the successful execution of AutoGrid, the genetic algorithm was set to default (the number of GA runs: 10; population size: 150; the number of energy evaluations: 2,500,000; and the number of generations: 27,000). The Lamarckian genetic algorithm was used, and the output was saved in a docking parameter file, DPF file format. After the generation of the DPF file, the AutoDock was executed. The input provided the AutoDock executable and DPF files and converted them to the docking log file (DLG). The generated result (in DLG format) was a log file that gave information, among others, about the top ten free binding energy and RMSD values by which SA binds to the amino acid. The results were analyzed from the analyze menu of the AutoDock tools; ranked based on their binding energies. The lowest binding energy complex was saved in PDBQT format for further analysis in the Protein–Ligand Interaction Profiler website (https://plip-tool.biotec.tu-dresden.de/plip-web/plip/index, accessed on 15 May 2021) and PyMOL software.

#### 4.3.7. Visualizing Interactions

The Protein–Ligand Interaction Profiler website and PyMOL 2.51 was used to visualize and study the 2-dimensional, 3-dimensional, and surface annotation of the SA’s interaction with the amino acids.

### 4.4. Identification of Sinapic Acid—Amino Acids Systems

#### 4.4.1. X-ray Powder Diffraction

The XRPD patterns were obtained using a Bruker AXS D2 Phaser diffractometer (Bruker, Germany) with CuKα radiation (1.54060 Å) and with a current of 10 mA and voltage of 30 kV. Data were collected at ambient temperatures, from 5° to 40° 2θ with a step size of 0.02° and a counting rate of 2 s·step^−1^. To analyze the acquired data, the Origin 2021b software (OriginLab Corporation, Northampton, MA, USA) was used.

#### 4.4.2. Scanning Electron Microscopy

Microscopic observations to study the particles’ morphology of the initial and ball milled, solvent evaporated, and freeze dried powders were performed using an Inspect S (FEI, Eindhoven, The Netherlands) scanning electron microscope.

#### 4.4.3. Fourier-Transform Infrared Spectroscopy

The FT-IR-ATR spectra were collected on an IRTracer-100 spectrophotometer. All spectra were measured in the mid-infrared area, over the range of 4000–400 cm^−1^ (400 scans at a resolution of 4 cm^−1^). Spectra were acquired and analyzed using the LabSolution IR software (version 1.86 SP2, Shimadzu, Kyoto, Japan). The results were interpreted by comparing the FT-IR peaks of pure substances with those of prepared systems.

The molecular geometries of SA and amino acids were optimized using the Density Functional Theory (DFT) method with Becke’s three-parameter hybrid functional (B3LYP), implemented with the standard 6-311G(d,p) as a basis set. Calculations of the normal mode frequencies and intensities were also performed. The PL-Grid platform (website: www.plgrid.pl, accessed on 11 March 2021) equipped with the Gaussian 09 package (Wallingford, CT USA) for DFT calculation was applicated. The GaussView (Wallingford, CT USA, Version E01) program was used to propose an initial geometry of investigated molecules and for visual inspection of the normal modes. The obtained data were analyzed using the Origin 2021b software (OriginLab Corporation, Northampton, MA, USA).

#### 4.4.4. Thermogravimetric and Differential Scanning Calorimetry

The TG analysis was performed using the TG 209 F3 Tarsus^®^ micro-thermobalance (Netzsch, Selb, Germany). About 6 mg powdered samples were placed in opened aluminum pans and heated at a scanning rate of 10 °C·min^−1^ from 25 to 500 °C in a nitrogen atmosphere, with a flow rate of 250 mL·min^−1^. The obtained TG data were analyzed using the Proteus 8.0 (Netzsch, Selb, Germany) software.

The DSC analysis was performed using the DSC 214 Polyma differential scanning calorimeter (Netzsch, Selb, Germany). A blank aluminum DSC pan was used as the reference sample. A total of 10 mg of SA was placed in the sealed pan with a hole in the lid. One heating mode (range 30–220 °C, 10 °C·min^−1^) was used to observe the melting point under a nitrogen atmosphere, with a flow rate of 250 mL·min^−1^. The obtained DSC data were analyzed using the Proteus 8.0 software (Netzsch, Selb, Germany). The visualization of the TG and DSC results was performed using the Origin 2021b software (OriginLab Corporation, Northampton, MA, USA).

### 4.5. Evaluation of Physicochemical Properties

#### 4.5.1. Physical Stability

The stability of the co-amorphous systems of SA with LYS were assessed by storing them in 30 °C and 50 °C in uncontrolled humidity in the laboratory incubator CLN 32 (Pol-eko Aparatura, Wodzisław Śląski, Poland), for six weeks. XRPD was utilized to identify the solid-state structures of the samples following a storage period of 2, 4, and 6 weeks.

#### 4.5.2. Chromatographic Conditions

The analysis was performed using a previously reported method [58], with modifications. The method was fully validated according to the ICH guidelines. The HPLC data were determined using Shimadzu Nexera (Shimadzu Corp., Kyoto, Japan) with the DAD detector. The SA determination was carried out using the Dr. Maisch ReproSil-Pur Basic-C18 column (250 mm × 4.6 mm; 5 µm; Dr. Maisch, Ammerbuch-Entringen, Germany) as the stationary phase and a methanol and 0.1% aqueous acetic acid solution (50:50, *v*/*v*) mixture as the mobile phase. The eluent flow rate was 1 mL·min^−1^. The detection wavelength was set at 323 nm, the injection volume was 1 µL, and the duration time of the method was 10 min. The retention time was 6.457 min. (Figure S7—Supplementary Materials).

#### 4.5.3. Solubility Study

The solubility of SA in the obtained systems, with LYS in molar ratio 1:1, was determined by weighing 24.78 mg of each sample and adding 5 mL of the medium (distilled water or HCl 0.1 N). Samples were agitated at 25 °C in a laboratory incubator MaxQ 4450 (Thermo Scientific, Waltham, MA, USA) at a speed of 75 rpm, for 2 h. The resultant solution was filtered through a 0.45 μm nylon membrane syringe filter. Measurements were performed in triplicate.

#### 4.5.4. Dissolution Rate Studies

The dissolution studies were performed in an apparatus with paddles. The samples containing 15 mg of pure SA and the SA-LYS co-amorphous systems, in an amount equivalent to 15 mg of SA, were added by sprinkling the powder on the surface of the distilled water or HCl 0.1 N (500 mL) as the dissolution medium, at a temperature of 37 °C, with paddles rotated at 75 rpm. At predetermined time intervals (5, 10, 15, 30, 45, 60, 90, 120, 180, and 240 min), 2 mL of the sample with the replacement of a pre-warmed dissolution medium was withdrawn and filtered through a 0.45 μm nylon membrane syringe filter. The amount of dissolved SA was analyzed using the developed and validated HPLC method.

### 4.6. Assessment of Biological Activity

#### 4.6.1. Antioxidant Activity Determination

##### CUPRAC Method

The SA antioxidant properties were determined by using the CUPRAC assay, according to Apak et al., with modifications [59]. The CUPRAC reagent was prepared from equal volumes of a 7.5 mM ethanolic 96% neocuproine solution, 10 mM CuCl_2_·H_2_O solution, and an ammonium acetate buffer of pH 7.0. The samples were prepared in the same way as for the solubility studies. A total of 50 µL of the sample was mixed with 150 µL of the CUPRAC reagent. The solution was incubated by shaking it in the dark, at an ambient temperature. After 30 min, absorbance was measured at the wavelength of 450 nm at the Multiskan GO plate reader (Thermo Fisher Scientific, Waltham, MA, USA). The results were presented as the IC_0.5_, which is the concentration at which the absorbance is equal to 0.5.

##### DPPH Method

The antioxidant activity of SA against the 2,2-diphenyl-1-picrylhydrazyl (DPPH) radical was performed on the basis of the method of Paczkowska-Walendowska et al., with modifications [60]. The samples were prepared in the same way as for the solubility studies. Briefly, 25 μL of samples were mixed with 175 μL of the 0.2 mM DPPH solution in a 96-well plate. The plate was protected from light, incubated, and shaken for 30 min at room temperature. Absorbance was measured at 517 nm against the control, which consisted of 25 μL of distilled water and 175 μL of the DPPH solution. The percentage of the radical scavenging activity was calculated with the use of the following Equation (1):

(1) $$Inhibition\;ability\left(\%\right)=\frac{Ao-Ai}{Ao}\times 100\%$$

where: *A*_o_ is the absorbance of the control; *A*_i_ is the absorbance of the samples.

### 4.7. Statistical Analysis

Statistical analyses were performed using Statistica 13.3 (StatSoft, Krakow, Poland). The data were analyzed using one-way analysis of variance (ANOVA) followed by the Duncan’s post-hoc test. A probability level of *p* < 0.05 was considered statistically significant. The results are reported as mean ± standard deviations.

## 5. Conclusions

As a result of theoretical and experimental studies, lysine was determined as the most effective co-former in obtaining co-amorphous systems with sinapic acid. Importantly, obtaining stable co-amorphous systems of sinapic acid with lysine was possible with the use of several preparation techniques (ball milling, solvent evaporation, and freeze drying). The physical stability of co-amorphous sinapic acid–lysine systems was determined by interactions that were confirmed at the molecular level. As a result of sinapic acid introduction as a part of co-amorphous systems, its solubility increased, which translated into improved antioxidant properties.

## Figures and Tables

**Table 1:** Summarized values of the binding energy for generated systems and possible interaction points between SA and amino acids.

Systems	SA–LYS	SA–TRP	SA–ARG	SA–HIS	SA–PRO
Binding Energy (kcal·Mol^−1^)	−1.71	−1.44	−1.21	−0.75	−0.57
Interaction SA–amino acid	O_2_–H_22_ O_3_–H_22_ O_4_–H_20_ H_28_–O_2_	H_28_–O_2_ O_4_–H_27_	O_2_–H_23_ O_3_–H_23_ H_28_–O_2_	O_4_–H_18_ H_28_–O_2_	O_4_–H_17_ H_28_–O_2_

**Table 2:** The results of solubility studies of SA and SA co-amorphous systems with LYS in molar ratio 1:1.

SA and Its Systems (Kind of Preparation)	Concentration (mg·mL^−1^)	
	Water	HCl 0.1 N
SA	0.226 ± 0.001 ^d^	0.209 ± 0.005 ^b^
SA-LYS 1:1 (ball milling)	2.914 ± 0.004 ^a^	0.313 ± 0.006 ^a^
SA-LYS 1:1 (solvent evaporation)	2.707 ± 0.001 ^b^	0.311 ± 0.006 ^a^
SA-LYS 1:1 (freeze drying)	2.498 ± 0.007 ^c^	0.303 ± 0.005 ^a^

The values are presented as the mean ± SD. “^a^” stands for the highest values, “^b^”, “^c^” and “^d^” stand for the statistically significant decreasing values.

**Table 3:** The results of SA amorphization with amino acids in each preparation techniques.

	LYS	ARG	TRP	PRO	HIS
Ball milling	✓	×	✓	×	×
Solvent evaporation	✓	✓	×	×	×
Freeze drying	✓	✓	×	×	×

The “✓” symbol indicates successful co-amorphization of SA with amino acids.

## Data Availability

Data available in a publicly accessible repository.

## Supplementary Materials

The supporting information can be downloaded at: https://www.mdpi.com/article/10.3390/ijms24065533/s1.
