# Supplementary material for: Sinapic Acid Co-Amorphous Systems with Amino Acids for Improved Solubility and Antioxidant Activity
Source: Int J Mol Sci. 2023 Mar 14;24(6):5533. doi: 10.3390/ijms24065533 (PMC10053217; doi:10.3390/ijms24065533)
Supplement: Supplementary file 1 [file ijms-24-05533-s001.zip › ijms-2232477-supplementary.pdf]

International Journal of *Molecular Sciences*

Supplementary materials

Sinapic Acid Co-Amorphous Systems with Amino Acids for Improved Solubility and Antioxidant Activity

Ewa Garbiec [1], Natalia Rosiak [1], Ewa Tykarska [2], Przemysław Zalewski[1] and Judyta Cielecka-Piontek [1,*]

[1] Department of Pharmacognosy, Poznan University of Medical Sciences, 3 Rokietnicka St., 60-806 Poznan, Poland; ewa.garbiec@student.ump.edu.pl (E.G.), nrosiak@ump.edu.pl (N.R.), pzalewski@ump.edu.pl (P.Z.),
[2] Department of Chemical Technology of Drugs, Poznan University of Medical Sciences, 6 Grunwaldzka St., 60-780 Poznan, Poland; etykarsk@ump.edu.pl
* Correspondence: jpiontek@ump.edu.pl

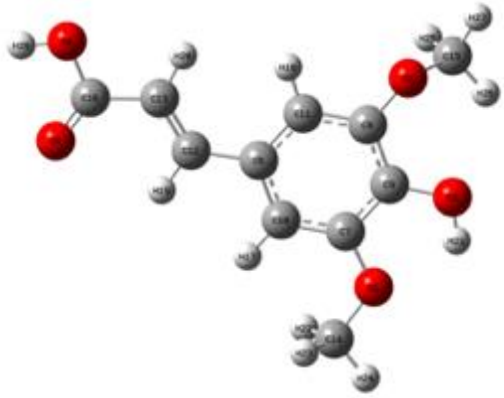

Optimized geometry of the SA.

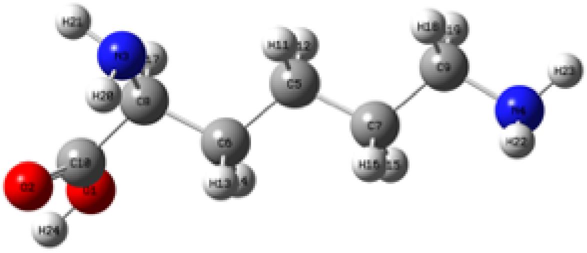

Optimized geometry of the LYS.

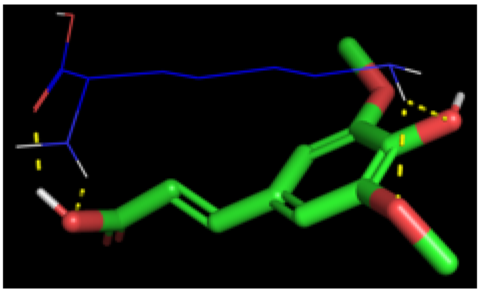

3D and 2D interaction of SA with LYS (−1.71 kcal·Mol^{-1}).

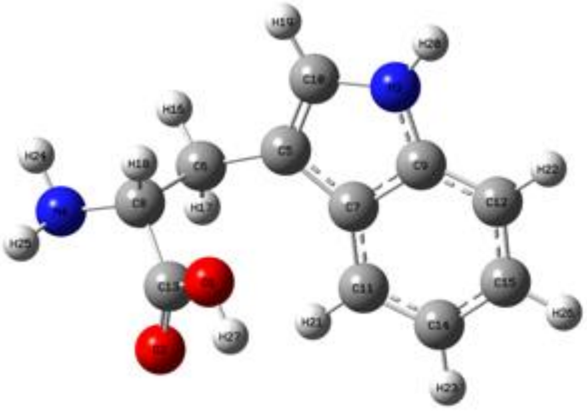

Optimized geometry of the TRP.

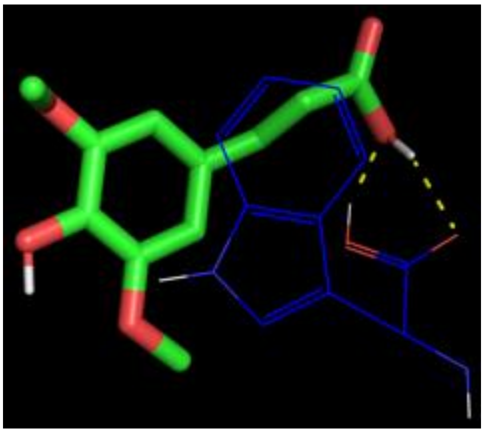

3D and 2D interaction of SA with TRP (−1.44 kcal·Mol^{-1}).

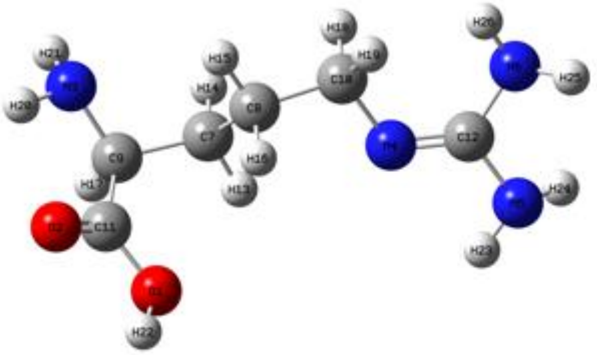

Optimized geometry of the ARG.

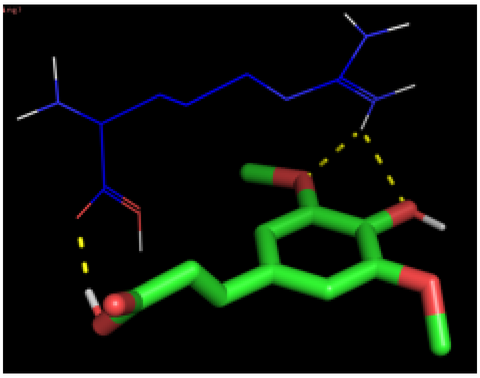

3D and 2D interaction of SA with ARG (−1.21 kcal·Mol^{-1}).

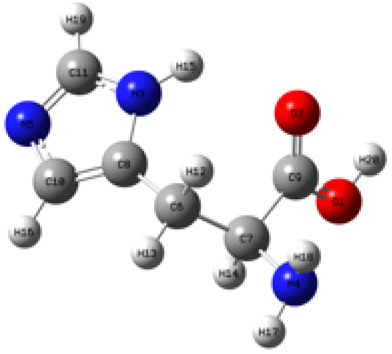

Optimized geometry of the HIS.

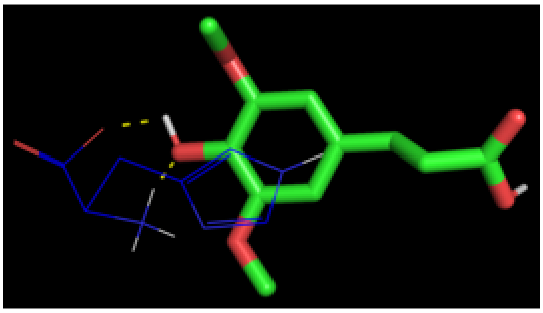

3D and 2D interaction of SA with HIS (−0.75 kcal·Mol^{-1}).

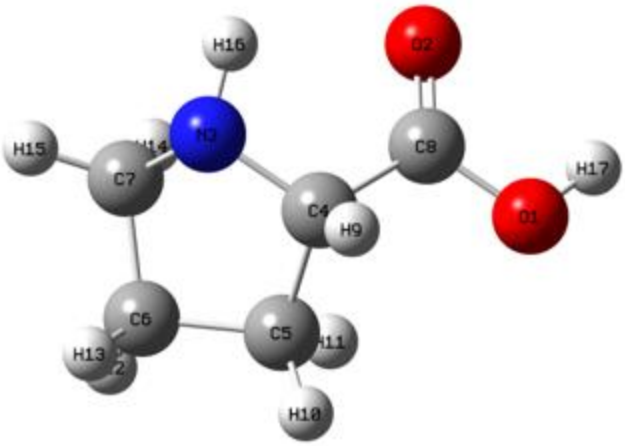

Optimized geometry of the PRO.

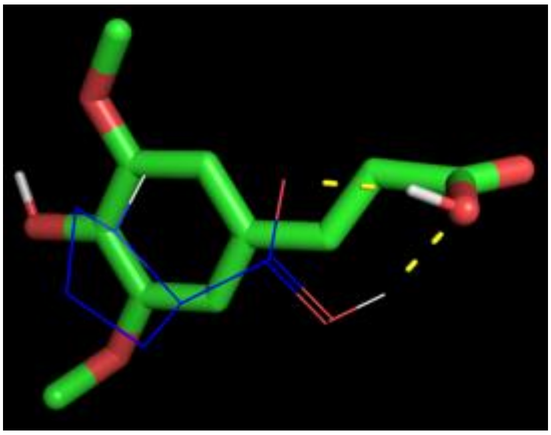

3D and 2D interaction of SA with PRO (−0.57 kcal·Mol^{-1}).

Figure S1. Optimized geometry of SA, LYS, TRP, ARG, HIS and PRO (images were generated with GaussView); and 3D and 2D interactions of SA with amino acids (images were generated with PyMol).

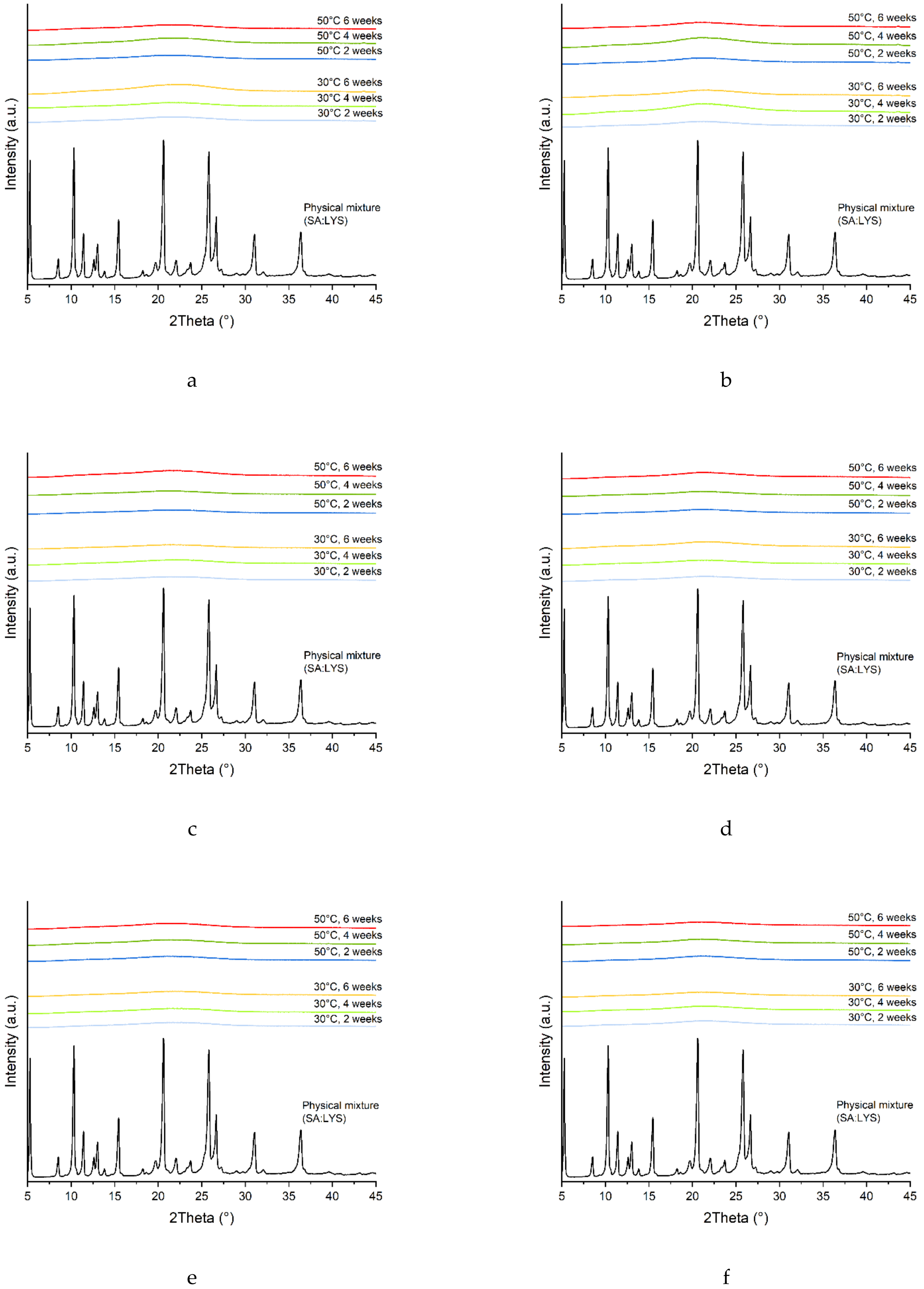

Figure S2. The XRPD diffractograms of stored SA-LYS systems at molar ratio 1:1 obtained by ball milling (a), at molar ratio 1:2 obtained by ball milling (b), at molar ratio 1:1 obtained by solvent evaporation (c), at molar ratio 1:2 obtained by solvent evaporation (d), at molar ratio 1:1 obtained by freeze-drying (e), at molar ratio 1:2 obtained by freeze-drying (f).

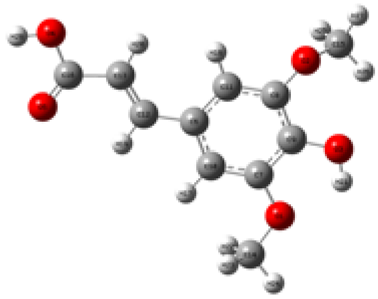

(a)

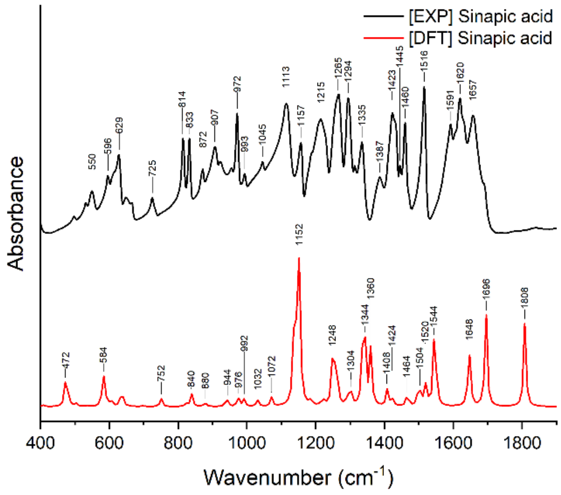

(b)

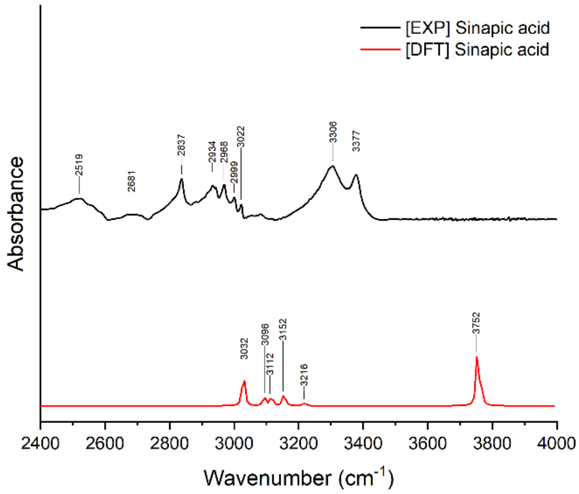

(c)

Figure S3. Optimized geometry of the SA (a); the FT-IR experimental results were compared with calculations obtained using basis set 6–311G(d,p); range 400–1900 cm^{-1} (b), range 2400–4000 cm^{-1} (c).

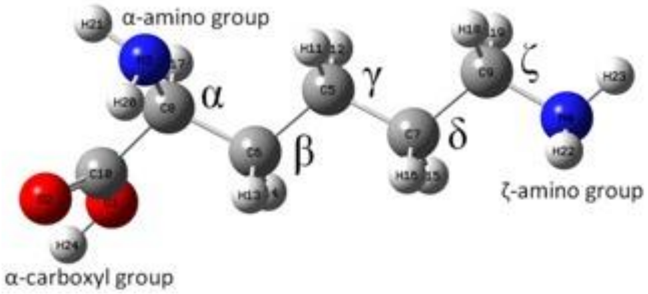

(a)

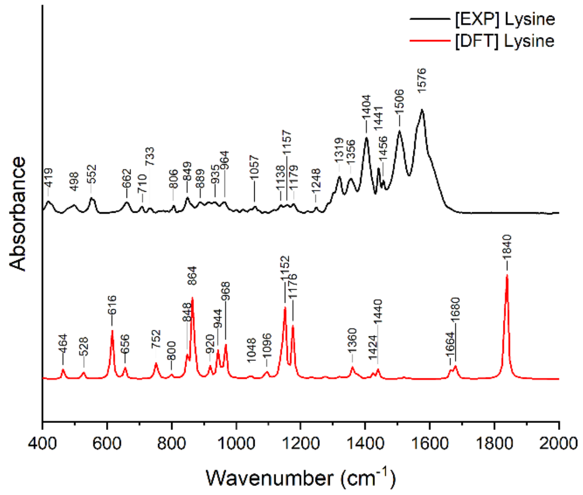

(b)

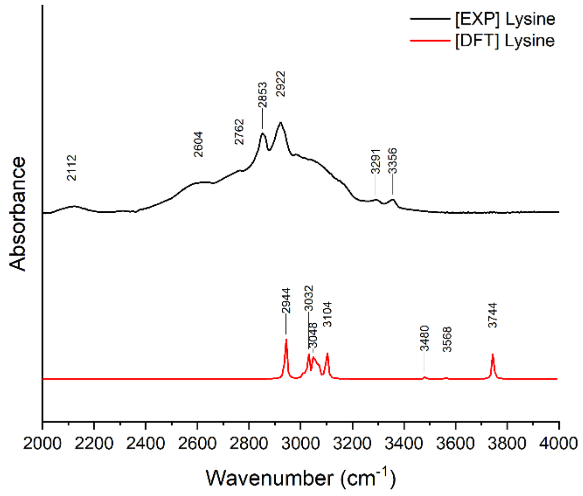

(c)

Figure S4. Optimized geometry of the LYS (a); the FT-IR experimental results were compared with calculations obtained using basis set 6–311G(d,p); range 400–2000 cm^{-1} (b), range 2000–4000 cm^{-1} (c).

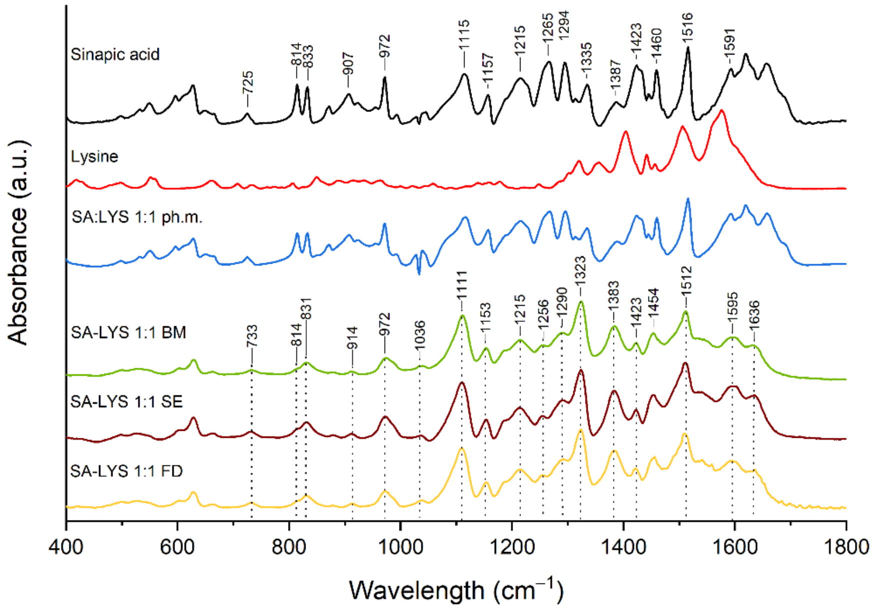

(a)

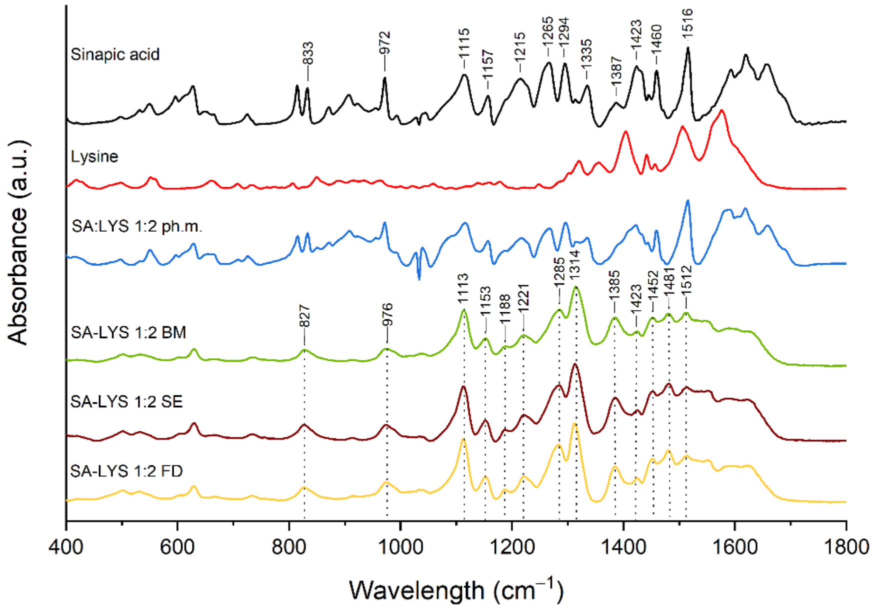

(b)

Figure S5. The FT-IR analysis: SA, LYS, SA:LYS 1:1 physical mixture, SA-LYS 1:1 ball milling; SA-LYS 1:1 solvent evaporation, SA-LYS 1:1 freeze drying (a); SA, LYS, SA:LYS 1:2 physical mixture, SA-LYS 1:2 ball milling; SA-LYS 1:2 solvent evaporation, SA-LYS 1:2 freeze drying (b); range 400–1800 cm^{-1}.

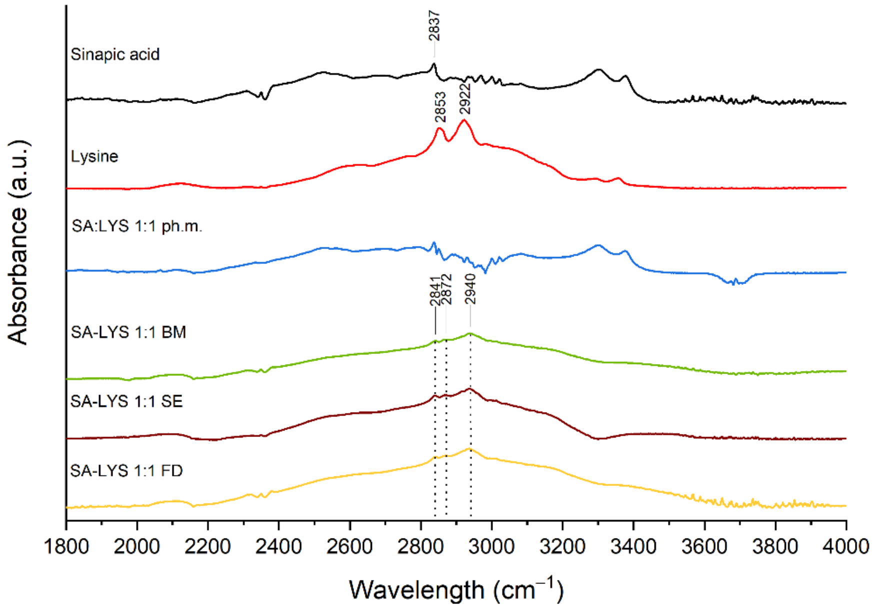

(a)

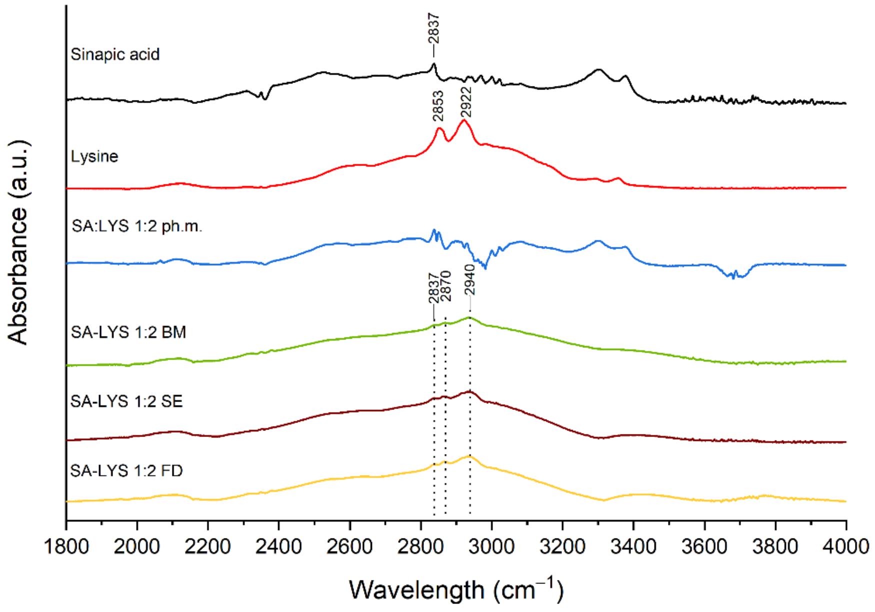

(b)

Figure S6. The FT-IR analysis: SA, LYS, SA:LYS 1:1 physical mixture, SA-LYS 1:1 ball milling; SA-LYS 1:1 solvent evaporation, SA-LYS 1:1 freeze drying (a); SA, LYS, SA:LYS 1:2 physical mixture, SA-LYS 1:2 ball milling; SA-LYS 1:2 solvent evaporation, SA-LYS 1:2 freeze drying (b); range 1800–4000 cm^{-1}.

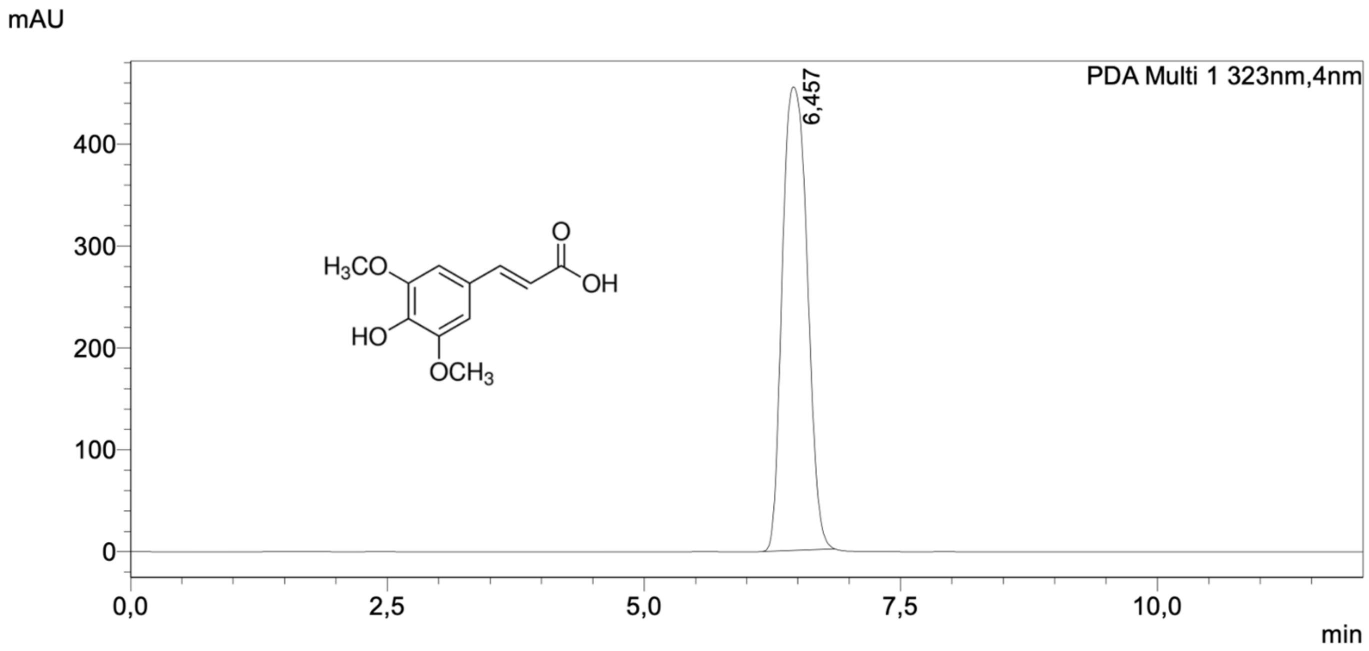

Figure S7. Chromatogram of SA for the developed method.

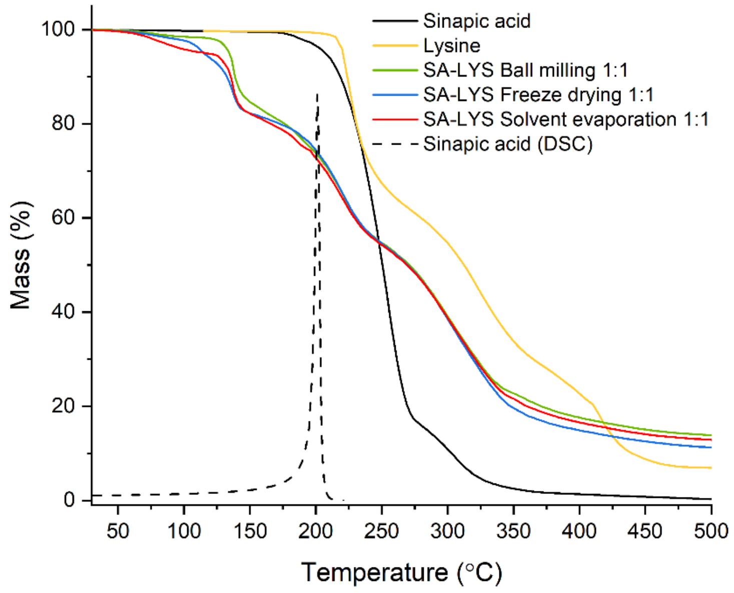

Figure S8. Thermogram of SA, LYS, and SA co-amorphous systems with LYS in molar ratio 1:1 obtained by ball milling, solvent evaporation, and freeze-drying.

Table S1. Selected characteristic bonds (in cm^{-1}) of SA, LYS, SA:LYS (ratio 1:1) physical mixture, co-amorphous system of SA-LYS (ratio 1:1), SA:LYS (ratio 1:2) physical mixture, co-amorphous system of SA-LYS (ratio 1:2). Legend: as-asymmetric, b-bending, r-rocking, s-stretching, t-twisting, w-wagging, def.-deformation, - - a band is not observed in this range

DFT	Sinapic acid	Lysine	SA-LYS 1-1	SA-LYS 1-2	Assignment
3752	3377		-		O–H s in carboxyl group
3568		3356	-		NH_2 as s in side chain
3752	3306		-		O–H s at phenyl ring
3480		3291	-		NH_2 s in amine group
3216	3022		-		C–H s
3152	2999		-		C–H s
3112	2968		-		C–H s
3096	2934		-		C–H as s
3104		2922	2940	2940	CH_2 as s at β-, γ- and δ-carbon
3048		2853	2872	2870	C–H s at γ-carbon + CH_2 as s at δ-carbon + C–H s at ζ-carbon
3032	2837		2841	2837	C–H s
3032		2762			CH_2 s at β-, γ- and δ-carbon
2944		2604			C–H s at ζ-carbon
1808	1657		-		C=O s + C–O–H b in carboxyl group + C=C s
1696	1620		1636		C=O s + C–O–H b in carboxyl group + C=C s + C–C–C as s in phenyl ring
1648	1591		1595		C–C–C b in phenyl ring + O–H b at phenyl ring
1840		1576			C=O s + C–O–H b in carboxyl group
1544	1516		1512	1512	C–O s in C–O–H + C–H r at phenyl ring + C–H_2 b + C–H w in methoxy group
1840		1506			C=O s + C–O–H b in carboxyl group
1520	1460		1454	1452	CH_2 b + C–H w in methoxy group
1680		1456			NH_2 b in amine group
1504	1445		-		CH_2 t + C–H r in methoxy group
1664		1441			NH_2 b in side chain
1464	1423		1423	1423	C–C–C s in phenyl ring + C–H w in methoxy group + O–H b at phenyl ring + C–H b
1440		1404			CH_2 w at δ-carbon + CH_2 w at ζ-carbon + NH_2 t in side chain
1424		1356			NH_2 t in amine group + C–H w at α-carbon + O–H b in carboxyl group
1424	1387		1383	1385	C–C–C as s in phenyl ring + O–H b at phenyl ring + C–H w in methoxy group
1408	1335		1323		C–C–C s in phenyl ring + C–H b + C–O–H b in carboxyl group
1360		1319			NH_2 t in amine group and side chain + O–H b in carboxyl group + CH_2 w at β- and δ-carbon + CH_2 t at γ- and ζ-carbon

1360	1294		1290	1314	deformation of phenyl ring + O–H b at phenyl ring and in carboxyl group + C–H b
1344	1267		1256	1285	breathing ring + O–H b at phenyl ring and in carboxyl group + C–H b
1176		1248			NH_2 w in amine group + O–H b in carboxyl group + C–H w at α-carbon + CH_2 t at β-, γ-, δ- and ζ-carbon
1304	1215		1215	1221	O–H b at phenyl ring and in carboxyl group + C–H b + C–C–C as s in phenyl ring
1152		1179			N–H w and N–H b in NH_2 in side chain + C–H w and C–H b in all side chain + O–H b in carboxyl group
1096		1157	1153		C–H w at α-carbon + CH_2 t at β- and γ-carbon + C–N s in side chain
1248	1157			1153	C–C–C as s in phenyl ring + O–H b at phenyl ring and in carboxyl group + C–H b at phenyl ring + C–H w in methoxy group
1048		1138			NH_2 t in all molecule
1152	1115		1111	1113	breathing ring + O–H b and C–O s in carboxyl group + C–H b
968		1057			β-carbon-γ-carbon-δ-carbon s + NH_2 t in side chain
1072	1045		1036		deformation of phenyl ring + C–H b at phenyl ring + C–O s
1032	993		-		C–H w
992	972		972		H–C=C r + C–C–C s in phenyl ring + C–O–C s
944		964			NH_2 w in amine group + NH_2 t in side chain + deformation side chain
976	907		914	972	breathing ring + C–C–O s + O–H b in carboxyl group + H–C=C r
920		889			NH_2 w in side chain + C–H r at β- and γ-carbon + C–H t at δ- and ζ-carbon
944	872		880		deformation of phenyl ring + C–O–C s in carboxyl group
864		849			NH_2 w in all molecule
880	833		831	827	C–H w
840	814		814		C–H w
848		806			NH_2 w and CH_2 r in all molecule +O–H b in carboxyl group
800		733			CH_2 r in all molecule
752	725		733		C–O–H w in carboxyl group + C–H w
752		710			C–O–H w in carboxyl group + CH_2 r at γ-, δ- and ζ-carbon
640	648				O–H w in carboxyl group + H–C–C t + C–C–H t
608	629				C–C–H t + O–H w in carboxyl group
584	596				breathing ring + O–H b and O–C=O b in carboxyl group + deformation all molecule
616		552			O–H w in carboxyl group
504	550		-		deformation all molecule
472	498		-		O–H b at phenyl ring
